# Demonstrating the successful application of synthetic learning in spine surgery for training multi–center models with increased patient privacy

**DOI:** 10.1038/s41598-023-39458-y

**Published:** 2023-08-01

**Authors:** Ethan Schonfeld, Anand Veeravagu

**Affiliations:** 1 Neurosurgery Artificial Intelligence Lab, Stanford University School of Medicine, Stanford, CA USA; 2 Department of Neurosurgery, Stanford University School of Medicine, Stanford, CA USA

**Keywords:** Machine learning, Spinal cord diseases

## Abstract

From real–time tumor classification to operative outcome prediction, applications of machine learning to neurosurgery are powerful. However, the translation of many of these applications are restricted by the lack of “big data” in neurosurgery. Important restrictions in patient privacy and sharing of imaging data reduce the diversity of the datasets used to train resulting models and therefore limit generalizability. Synthetic learning is a recent development in machine learning that generates synthetic data from real data and uses the synthetic data to train downstream models while preserving patient privacy. Such an approach has yet to be successfully demonstrated in the spine surgery domain. Spine radiographs were collected from the VinDR–SpineXR dataset, with 1470 labeled as abnormal and 2303 labeled as normal. A conditional generative adversarial network (GAN) was trained on the radiographs to generate a spine radiograph and normal/abnormal label. A modified conditional GAN (SpineGAN) was trained on the same task. A convolutional neural network (CNN) was trained using the real data to label abnormal radiographs. A CNN was trained to label abnormal radiographs using synthetic images from the GAN and in a separate experiment from SpineGAN. Using the real radiographs, an AUC of 0.856 was achieved in abnormality classification. Training on synthetic data generated by the standard GAN (AUC of 0.814) and synthetic data generated by our SpineGAN (AUC of 0.830) resulted in similar classifier performance. SpineGAN generated images with higher FID and lower precision scores, but with higher recall and increased performance when used for synthetic learning. The successful application of synthetic learning was demonstrated in the spine surgery domain for the classification of spine radiographs as abnormal or normal. A modified domain–relevant GAN is introduced for the generation of spine images, evidencing the importance of domain–relevant generation techniques in synthetic learning. Synthetic learning can allow neurosurgery to use larger and more diverse patient imaging sets to train more generalizable algorithms with greater patient privacy.

## Introduction

Machine learning (ML) has already made significant impacts on neurosurgery. Such impacts include advances in surgical planning and outcome prediction^1–4^, neuronavigation^5,6^, and robotics^7–11^. A 2020 global survey found that 28.5% of neurosurgeons use ML in their clinical practice, most commonly applied for outcome prediction and interpretation of imaging^12^. However, for most desired applications of ML in neurosurgery that requires complex data types beyond those found in large claims datasets, there has been limited progress. This is due to the lack of available, diverse “big” data in neurosurgery. Until 2021, the largest publicly available dataset in the spine domain contained only 797 images labeled with only the spine position^13^. In 2018, only 60 images^13^. A major identified barrier to “big data” in neurosurgery is that of data ownership and exchange where providers and systems are hesitant to share data largely motivated by legal and data privacy considerations^14^.

Such privacy preservation prohibits the sharing of medical data across institutions which not only restricts the ability to build large datasets, required for the training of most deep learning models, but also restricts algorithms to learn from data from largely only one institution^15^. This “domain shift” in turn results in internally validated algorithms failing to generalize and externally validate^16^. One possible solution is to share synthetic data that represents the clinical information of patient data but preserves its privacy; state-of-the-art efforts have begun to use GANs to generate this synthetic data and thereby allow for its release and sharing across institutions. Synthetic Learning (SL) is unbiased and downstream models trained on the synthetic data have achieved high performance on brain tumor^17^ and nuclei segmentation^17^ tasks.

It is imperative to determine the success of SL in a domain specific method. The generation of certain data types may be possible in one neurosurgical domain but not the other. Furthermore, while generation of synthetic data may be possible, successful training of downstream ML models on the synthetic data in certain neurosurgical subdomain and subtasks may be impossible. Therefore, it is imperative to determine the success of SL in a domain specific method before large, expensive efforts commit to use the technique to build towards “big data” in neurosurgery. While a federated learning (FL) study has demonstrated vertebral body segmentation^18^, we could not find any study of SL in the spine domain. Furthermore, all the aforementioned demonstrations of SL in neurosurgery have only been validated on physical tasks of segmentation and not clinical tasks such as imaging analysis. In this study we seek to study the application of generative SL in the spine domain and validate the method’s success for the first time on a clinical analysis task of determining image abnormality from spine radiographs.

## Methods

### Data source

VinDR-SpineXR dataset has 10,466 spine radiographs that are annotated for 13 different classifications with respective bounding boxes^13^. These radiographs, coming from the Hanoi Medical University Hospital, were labeled for the 13 lesions by a committee of three experienced radiologists. Example classifications are enthesophytes, vertebral collapse, or spondylolisthesis. Each of the 5000 studies included in the dataset was labeled by one of the three radiologists. For each imaging study, information on the presence of an abnormality, the classification of the abnormality, bounding boxes of each abnormality, and basic demographic information of the patient is provided.

### Variables and outcomes

For each imaging study, information on the presence of an abnormality, the classification of the abnormality, bounding boxes of each abnormality, and basic demographic information of the patient is provided.

The primary outcome of the study was the binary prediction of a radiograph being abnormal or normal using a machine learning model trained only using synthetic “fake” data.

### Data pre–processing

After data pre–processing there were 1470 abnormal radiographs and 2303 normal radiographs to be included in the study which were split 80–20 into a training and testing set. Due to high computational cost of training the GAN and SpineGAN, and to maximize the size of training sets, no validation set was used to determine hyperparameters. Instead, hyperparameters were selected from reported literature and the standard NVIDEA implementation of StyleGAN2 with Adaptive Discriminator Augmentation^19,20^.

Each image is in DICOM format with some identifying information removed. The pixel arrays of the DICOM images are grayscale and of varying dimensions and resolutions. Because DICOMS were identified, many are missing information, some even missing pixel arrays, and thus require serious preprocessing. Each DICOM was preprocessed to extract its pixel array and its abnormality status using the image ID. To further preprocess each pixel array, the smallest dimension was taken of the image, and a square, centered at the image’s center was cropped for final use from the pixel array. Any image with the smallest dimension less than 128 pixels was not used for model training. Finally, all square cropped images were downsampled using a nearest neighbors approach to 256 by 256 resolution. After preprocessing, there were 1470 abnormal radiographs and 2303 normal radiographs to be used for all later model training.

All training was done on a google cloud virtual machine using 1 × NVIDIA Tesla A100. Data augmentation for the classification models included a random horizontal flip with 0.3 probability, a random rotation from − 5 to 5°, and a random resized crop (scale = 0.8, 1.0) and (1.0, 1.0) ratio. Preprocessing for all classification models included a resize to 224 square pixels, and normalization to (mean = [0.485, 0.456, 0.406], std = [0.229, 0.224, 0.225]).

### Generating synthetic data

**Figure 1:**
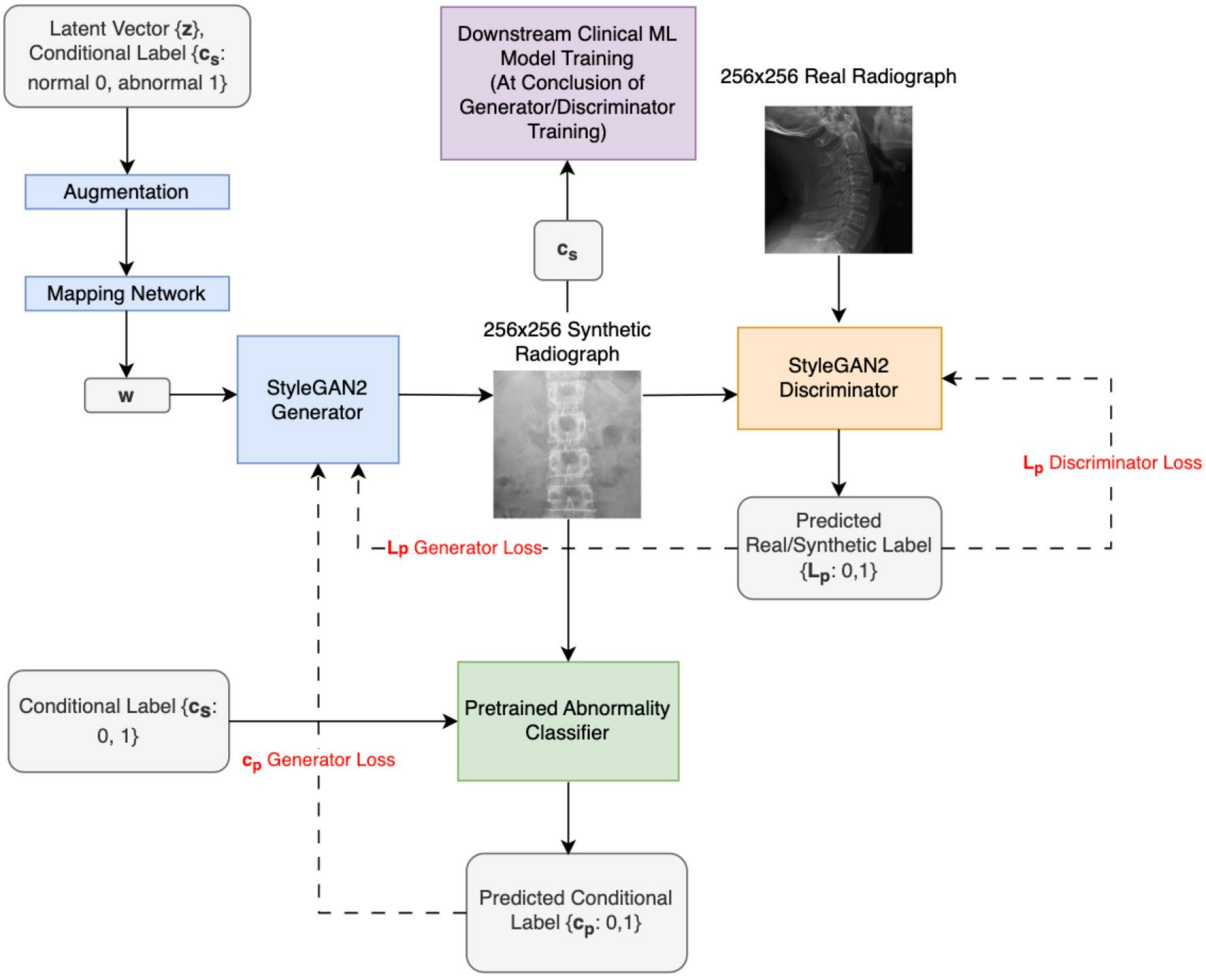
Architecture SpineGAN generation of synthetic images and training of downstream machine learning models. The StyleGAN2 Generator architecture is given as a blue box in the figure and fully detailed in the original publication (Karras et al.)^19^ as is the StyleGAN2 Discriminator architecture with the orange box (Karras et al.)^19^. The SpineGAN modification to StyleGAN2 network is to introduce a pre-trained abnormality classifier to supplement the generator’s loss term with c_p_ in addition to the standard L_p_ loss from the discriminator output. All loss terms are represented in red.

To generate synthetic spine radiographs from the real spine radiographs, a generative adversarial network (GAN) was used (Fig. 1). A GAN uses two competing deep neural networks (DNN), one that attempts to generate synthetic data, and one that competes to determine if data is from the real training set or is synthetic. By alternating the training of the two DNNs, both improve, and the generative DNN may be used to generate synthetic data. The GAN was made to be conditional such that after training, it could generate an abnormal spine radiograph when prompted, or a normal radiograph when prompted. While most GANs take as input a vector of random noise to generator a unique image, the GANs trained in this work take an additional input of the desired class of image to generate (ie. normal, abnormal).

To improve the generation of synthetic data, we introduce a novel GAN method that first trains a classifier on the real data to classify abnormal spine radiographs (Fig. 1). Then, when training the two competing DNNs, both the score from the competing DNN determining if the image is real or synthetic, and the score from the trained classifier determining if the image is clinically abnormal or normal is passed back as feedback to the generative model. The purpose of this novel GAN, which we call SpineGAN, was to introduce clinical information during GAN training to generate better labeled synthetic images. The novel loss term for the SpineGAN generator is included below:

The first line of the loss formula represents the standard loss term for the generator of SpineGAN. The second line represents the novel term that is a standard cross entropy loss for a classifier which represents whether the conditional label matches the predicted label by the pretrained network S.

1. Generator Loss Function (Gℓ): This term represents the loss function used to train the generator network. It measures the discrepancy between the generated samples (obtained by applying the generator network to a vector of class labels) and the discriminator's prediction. The goal is to minimize this loss, which encourages the generator to produce samples that are indistinguishable from real data.
2. Vector Obtained by the Generator Network ($\overrightarrow{x}$): It refers to the output vector generated by the generator network when provided with a vector of class labels ($\overrightarrow{c}$) for a mini-batch of size m. This vector represents the synthetic data samples created by the generator.
3. Discriminator Network (D): The discriminator network is responsible for distinguishing between real data and the generated samples. It takes a data sample (either real or generated) as input and produces a probability score using the sigmoid activation function (σ). This score indicates the likelihood of the input being real.
4. Sigmoid Activation Function (σ): The sigmoid activation function is a mathematical function that maps the input values to a range between 0 and 1. In the context of the discriminator network, it is used to squash the output into a probability score, where values closer to 1 indicate a higher likelihood of the input being real.
5. Regularization Term (γ): This term represents a regularization parameter that is used to control the influence of regularization in the loss function. Regularization helps prevent overfitting and encourages the model to generalize well to unseen data. By adjusting the value of γ, you can control the impact of regularization on the overall loss.
6. Class Label (c_i_): It refers to the class label assigned to the ith element in the mini-batch. In the context of the generator network, these class labels are used as input to generate samples corresponding to specific classes (normal or abnormal).
7. Output of the Pretrained Abnormality Classification Network S ($S(\overrightarrow{x}i)$): This term represents the output of a pre-existing abnormality classification network, denoted as S. It takes the ith element in the mini-batch ($\overrightarrow{x}i$) as input and produces a numerical value, which represents the abnormality classification score for that element. The value can be interpreted as the likelihood of abnormality in the input.
  $${\text{SpineGAN}}\left( {{\text{G}}\ell } \right){ = } - {\text{log}}\sigma \left( {D\left( {\vec{x},\vec{c}} \right)} \right) + \gamma \mathop \sum \limits_{i = 1}^{m} ci{\text{ log}}S\left( {\vec{x}i} \right) - \gamma \mathop \sum \limits_{i = 1}^{m} \left( {1 - ci} \right){\text{log}}\left( {1 - S\left( {\vec{x}i} \right)} \right)$$

Lastly, a multi–conditional GAN was trained to generate spine radiographs with a detailed abnormality label on command. These abnormality labels included: No finding, Disc space narrowing, Foraminal stenosis, Osteophytes, Spondylolisthesis, Surgical implant, Vertebral collapse, Other lesions. The purpose of this multi–conditional GAN was to provide preliminary evidence that generating synthetic data in spine surgery domain applies to more complex tasks than binary classification. The training details and hyperparameters for the multi–conditional GAN were the same as for the conditional (normal/abnormal) GAN and SpineGAN. However, due to computational cost, the multi–conditional GAN was only trained for 1,200,000 images shown to the discriminator, where for GAN and SpineGAN, 4,200,000 images were shown to the discriminator. Therefore, the multi-conditional GAN was used as proof of concept of the ability to generate abnormality conditional synthetic spine radiographs. Please see Supplemental Methods for more information regarding GAN training.

### Classifier models

A convolutional neural network (CNN) was trained on the real radiographs and evaluated on the test set of the real radiographs to determine the ability for abnormality binary classification using the real data. Then, in two experiments, using 10,000 synthetic images generated either by the GAN or SpineGAN, the same CNN was trained on the synthetic images and tested on the real data test set to evaluate the performance of synthetic learning. The Adam optimizer with learning rate 0.001 and batch size of 32 was used to train all classifiers. Please see Supplemental Methods for more information regarding classifier model training.

### Performance metrics

Frechet´ Inception Distance (FID), Precision, and Recall metrics were computed to evaluate the generative ability of the GAN both during training and at the completion of training. Lower FID indicates that the synthetic images appear closer to the real images, and thus lower FID metric indicates improved GAN training. Higher precision similarly evidences improved GAN training, and higher recall indicates that the synthetic images generated are more diverse. The Precision metric calculates the probability that a random generated image falls within the support of the real image distribution. The Recall metric calculates the probability that a random real image falls within the support of the generated image distribution. Both precision and recall were calculated according to the NVIDEA implementation of StyleGan2^19,20^ and are formulaically developed and defined in Sajjadi et al.^21^. Roughly, to evaluate whether an image falls within the real image distribution, one determines if the image’s embedding is within the space containing the embedding of the real images.

## Results

### Predicting abnormality classification using real radiographs

A CNN was trained on real radiographs to predict whether each radiograph was abnormal or normal. The CNN achieved an AUC of 0.856 which shows similar performance to the state-of-the-art performance on this task reported by Nguyen et al. as 0.886^13^ (Table 1).

**Table 1:** Classifier performance on held–out test set.

Abnormality classification	Test AUC
Nyugen et al. (State of the art) ^13^	0.886
Real data	0.856
Synthetic data (GAN)	0.814
Synthetic data (SpineGAN)	0.830

Comparing state–of–the–art trained classifier with classifiers trained on the real data, synthetic data from GAN, and synthetic data from SpineGAN.

### Generating synthetic data

**Figure 2:**
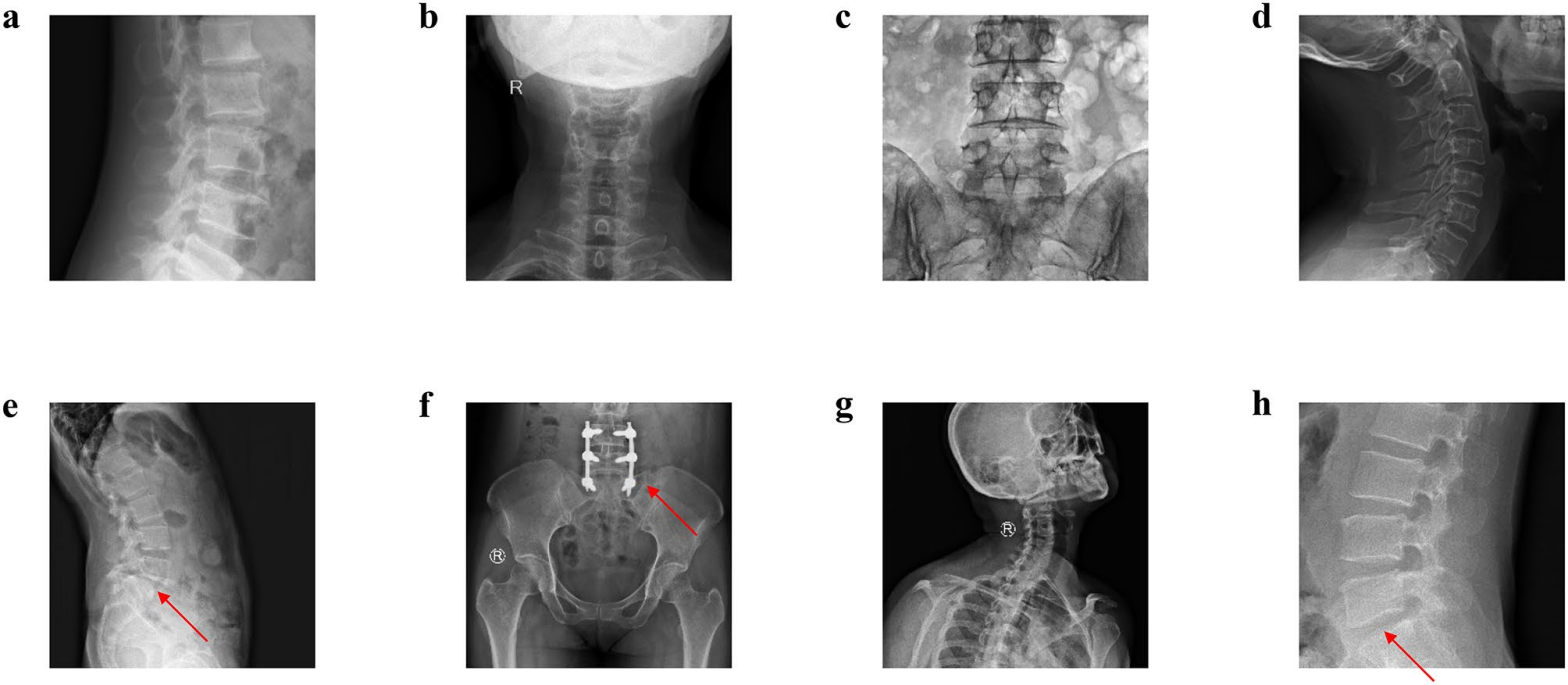
Real spine radiographs used to train the abnormality classifier on real data and train the generative models (GAN and SpineGAN), (**a**–**d**, **g**) No finding, (**e**) spondylolisthesis, (**f**) surgical implant), (**h**) disc space narrowing.

**Figure 3:**
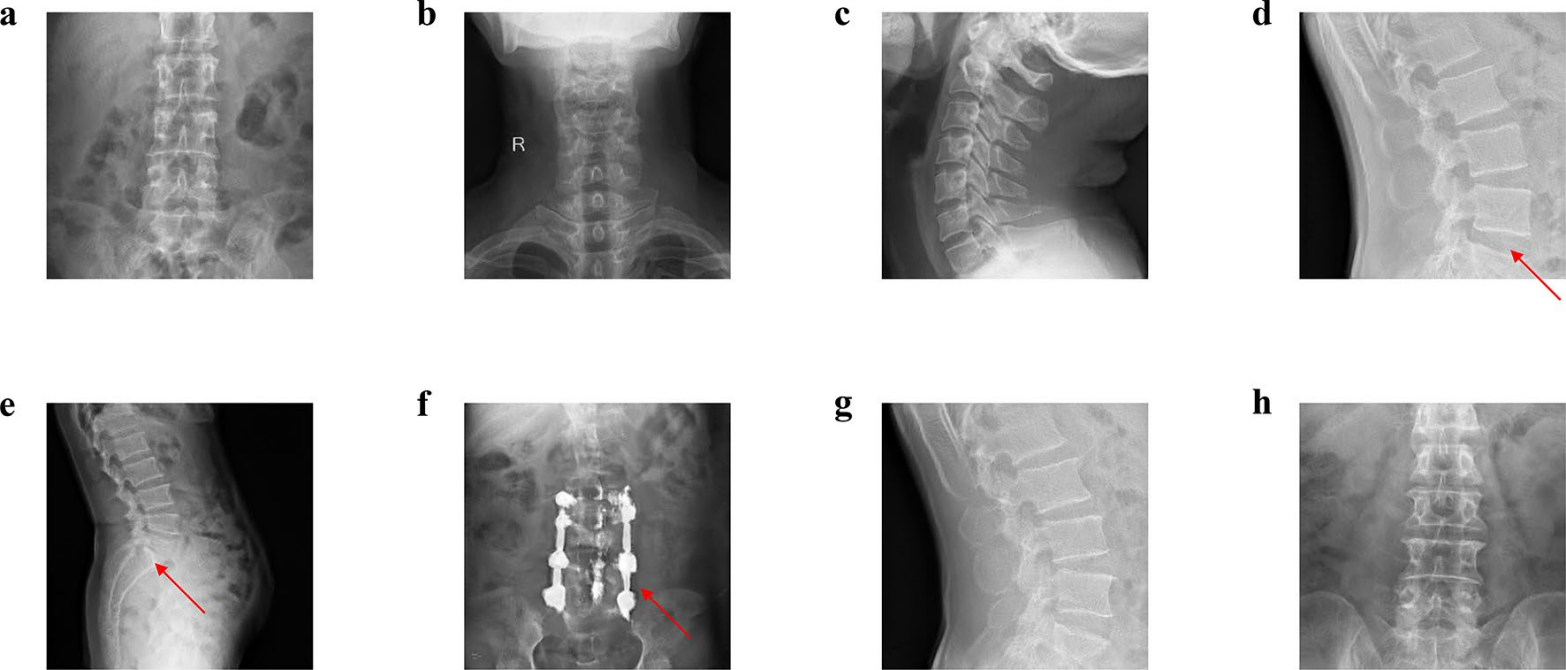
GAN generated synthetic spine radiographs specified to produce normal radiographs (**a**–**d**) and abnormal radiographs (**e**–**h**), (**a**–**c**, **g**–**h**) no finding, (**d**) disc space narrowing, (**e**) spondylolisthesis, (**f**) surgical implant.

**Figure 4:**
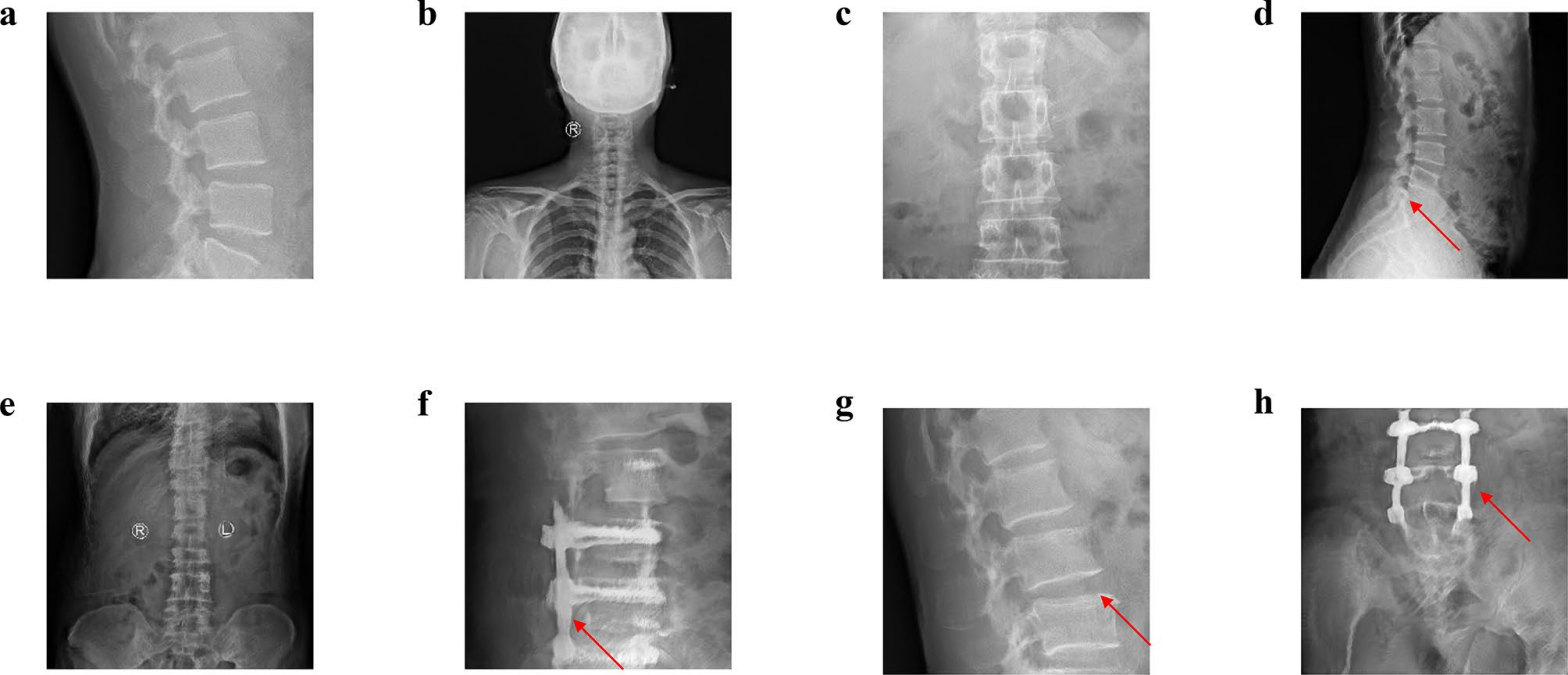
SpineGAN generated synthetic spine radiographs specified to produce normal radiographs (**a**–**d**) and abnormal radiographs (**e**–**h**), (**a**–**c**, **e**) no finding, (**d**) spondylolisthesis, (**f**) surgical implant, (**g**) disc space narrowing, (**h**) surgical implant.

Both a GAN and a domain relevant SpineGAN were trained to each generate synthetic radiographs with a corresponding label of either abnormal or normal. The real data, comprised of both normal and abnormal labeled spine radiographs, is shown in Fig. 2. Figures 3 and 4 demonstrate the successful generation of synthetic data by a classical GAN and SpineGAN respectively. The quality of the spine radiographs generated with a normal label (Figs. 3a–d, 4a–d) does not seem to markedly differ from the quality of those generated with an abnormal label (Figs. 3e–h, 4e–h). From Figs. 3f and 4f, h, both the GAN and SpineGAN learned to generate abnormal radiographs that had a surgical implant.

From Table 2, both the GAN and SpineGAN achieved high scores on generative metrics, with the GAN having superior scores on FID and Precision (FID: 32.7, Precision: 0.460) as compared to SpineGAN (FID: 33.9, Precision: 0.418), and SpineGAN having a superior score for Recall (0.0194) compared to that of the GAN (0.0159).

**Table 2:** Quantitative metrics (Fréchet Inception Distance, Precision, Recall) are provided for the GAN and SpineGAN generated synthetic spine radiographs.

Generative metric analysis	FID	Precision	Recall
GAN	32.7	0.460	0.0159
SpineGAN	33.9	0.418	0.0914

### Predicting abnormality classification using synthetic radiographs

A CNN was trained on synthetic radiographs to predict the binary task of abnormal or normal. When trained on synthetic images from the GAN, the CNN achieved an AUC of 0.814, while the CNN that was trained on synthetic images from SpineGAN achieved an AUC of 0.830 (Table 1).

### Generating multi–conditional synthetic data

**Figure 5:**
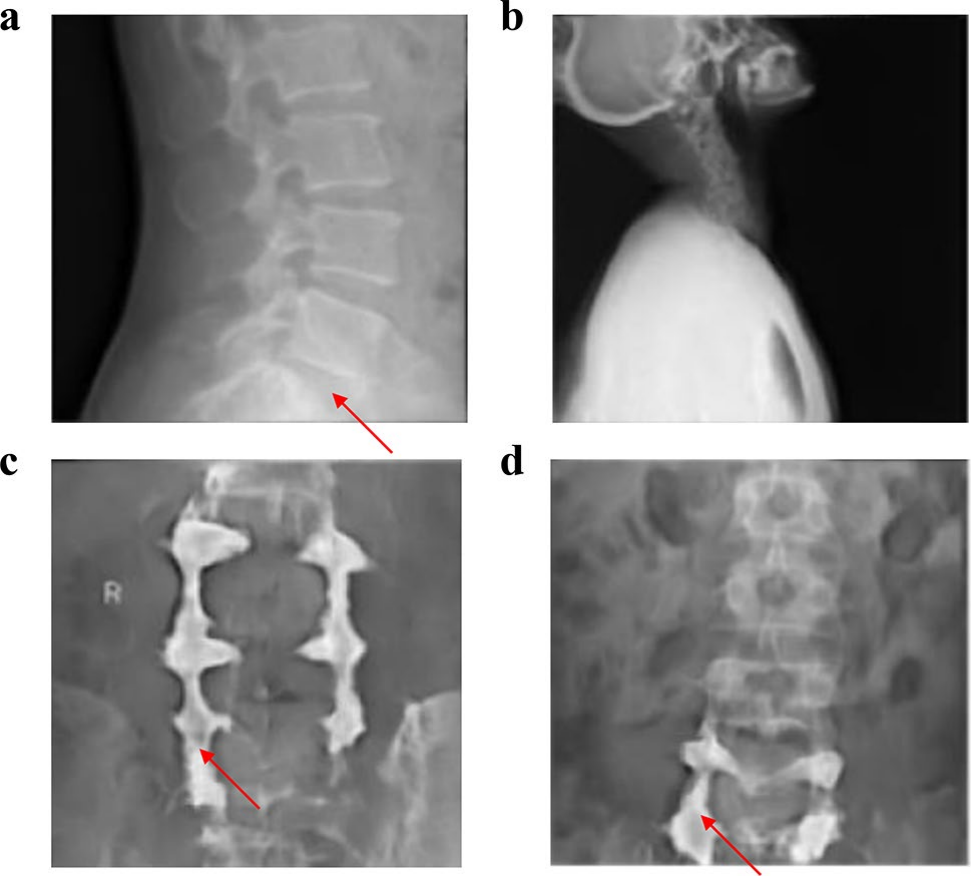
Selected examples from the proof-of-concept conditional generation of synthetic spine radiographs with a specific abnormality label options: No finding, Disc space narrowing, Foraminal stenosis, Osteophytes, Spondylolisthesis, Surgical implant, Vertebral collapse, Other lesions. (**a**) disc space narrowing, (**b**) no finding, (**c**–**d**) surgical implant.

Examples from the successful proof of concept of generating synthetic spine radiographs with specific abnormalities are shown in Fig. 5a–d. In the four examples provided, both spondylolisthesis and surgical implant are visualized.

## Discussion

Using spine radiographs labeled as either normal or abnormal, we conditionally generated synthetic spine radiographs that were respectively labeled. We demonstrate both the successful generation of synthetic spine imaging and successful training of a machine learning classifier on the synthetic data with similar performance to a classifier trained on the real data. This represents the first time that synthetic learning (SL) has been demonstrated in the domain of spine surgery and the first time that SL has been demonstrated in neurosurgery to work for imaging analysis. These results have immediate clinical application for efforts seeking to release patient data to the research community without privacy concerns. By generating and sharing synthetic data using the approach outlined in this work, institutions can together build generalizable, big datasets of clinically meaningful imaging information, with confidence from our results of the successful ability to train machine learning models on the synthetic data with similar performance. Furthermore, we introduce a domain specific method to generate the synthetic data, that we demonstrate results in greater clinical information generated with improved ability to train downstream machine learning models. This method is outlined and can be adopted for all future applications of synthetic learning in neurosurgery and other medical domains.

Evidence of the successful generation of spine radiographs by both a traditional GAN and a modified domain relevant GAN that we term SpineGAN is provided by quantitative metrics. Both generative models achieved very strong FID scores that measure the quality of the generated images (Table 2) as well as high precision scores (Table 2). As both of these metrics are domain agnostic, increased confidence of the successful generative quality is provided by the success of training the downstream abnormality classifier (Table 1). For generative ML, evaluating the quality of generations suffers from a lack of metrics that consider medical factors. Instead, qualitative analysis of generated examples is crucial. For both the GAN (Fig. 3) and SpineGAN (Fig. 4), both normal and abnormal generated radiographs resemble the real radiographs (Fig. 2) in their diversity of beam orientation, levels, and abnormality types. As a proof of concept for the success of SL for more clinically complex tasks, a multi–conditional GAN to generate specific abnormalities on spine radiographs was trained for the project. Despite limited training due to high computational costs of the project, successful generation was achieved (Fig. 5).

A key element of this work is the development of a modified GAN for the task of SL in spine domain. SpineGAN relies on a preliminary trained model that can differentiate normal and abnormal radiographs to be used during its training. While traditional GANs are trained with feedback on whether the fake image was deemed real, SpineGAN trained with additional feedback on whether the fake image’s label was deemed correct by the classifier. Motivation for this approach was to incorporate domain specific information during training time to push the generative training towards clinically relevant differences in its generations, for the success of training downstream models on the synthetic data. From Supplementary Fig. 1, SpineGAN not only achieved similar performance on traditional generative metrics as the GAN, but it had higher recall likely corresponding to more clinically diverse images and trained faster. This faster training is an important consideration as these models take multiple days and hundreds of dollars to train. Finally, SpineGAN achieved an AUC of 0.830 compared to an AUC of 0.814 by the traditional GAN for training the SL classifier (Table 1). Considering the AUC of 0.856 using real data, SpineGAN more closely preserves clinical information in its synthetic generations compared to the traditional GAN.

Preserving data privacy while building datasets representative of multiple centers is currently a major topic of discussion in neurosurgery^22^. The most common approach to this problem, Federated Learning (FL), trains an ML model at each institution and then aggregates these models without any data ever leaving each institution. FL has been successfully demonstrated for the detection of intracranial hemorrhage^22^, vertebral body segmentation^18^, and tumor boundary detection for glioblastoma^23^. However, there are two problems with FL. The first problem is that recent work has demonstrated that computational attacks can rederive the patient data used in FL^24^. The second problem is that once the FL algorithm is trained, it is impossible to use the data across the institutions to train a similar ML model for another purpose without starting over. With SL as demonstrated in this work for spine surgery, the research community benefits from a dataset of synthetic images that can be used and reused to train ML models.

As the synthetic learning strategy presented in this work is expanded to more complex downstream machine learning tasks or to generating synthetic scans to be used for education, it likely will be important to have a method to filter bad–quality synthetic images. Here we propose a few such methods. The most traditional method is easily accomplishable from the current code release. For each generated image, a noise vector is passed as input alongside the conditional label (normal/abnormal). These noise vectors come from a probability distribution, where the generative model produces more standard images from noise vectors that are closer to the center of the distribution. To encourage high–quality images, the noise vectors used for input can be thresholded to only use those that come from within a selected area near the center of the distribution, depending on the downstream task. However, while this is currently feasible and highly scalable, it is not a full guarantee of high quality. Other strategies include using a downstream classifier that can classify an image as being a spine radiograph or not and filtering out any synthetic images that do not pass this test.

Despite the findings, the study has several limitations. Firstly, the SL approach used requires high technical capability and computational costs (~ 500 USD) to train the model used to generate the synthetic data. Because SL has immediate clinical applications, but a large technical barrier, a simple package was developed that can be used to replicate the method on new real data with the only prerequisite of very basic coding experience. This repository is publicly available (https://github.com/Ethan-schonfeld/SpineGAN) and future work can develop the provided code into a clinically accessible application. Another limitation of the work is that while the SL approach was validated on abnormality classification, it may not apply for more complex image analysis such as benign and malignant diagnosis of spinal tumors by ML on MRI^25^. This work is the first demonstration of the success of SL on any neurosurgical clinical image analysis beyond structural segmentation and future work will investigate SL on more complex analysis.

## Conclusion

The successful application of synthetic learning was demonstrated in the spine surgery domain for the classification of spine radiographs as abnormal or normal. For the first time in neurosurgery, synthetic learning was demonstrated for a clinical image analysis task. A modified domain–relevant GAN is introduced for the generation of spine images, evidencing the importance of domain–relevant generation techniques in synthetic learning. Synthetic learning can allow neurosurgery to use larger and more diverse patient imaging sets to train more generalizable algorithms with greater patient privacy.

## Supplementary Information

Supplementary Information 1.

Supplementary Information 2.

## Data Availability

The datasets generated and/or analysed during the current study are available in the VinDR-SpineXR PhysioNet repository, https://www.physionet.org/content/vindrspinexr/1.0.0/. Please contact ethan.schonfeld@stanford.edu for more information.
