# Supplementary material for: Demonstrating the successful application of synthetic learning in spine surgery for training multi–center models with increased patient privacy
Source: Sci Rep. 2023 Aug 1;13:12481. doi: 10.1038/s41598-023-39458-y (PMC10393976; doi:10.1038/s41598-023-39458-y)
Supplement: Supplementary file 1 — Supplementary Information 1. [file 41598_2023_39458_MOESM1_ESM.docx]

*Generating Synthetic Data*

All GAN models used the architecture of StyleGAN2–ADA as adaptive discriminator augmentation was found to improve generative quality for small sized training datasets. StyleGAN2–ADA was made to be a conditional GAN for 2 classes: normal spine radiographs and abnormal spine radiographs. StyleGAN–ADA– MultiConditional was trained to be conditional with eight classes of radiographs: No finding, Disc space narrowing, Foraminal stenosis, Osteophytes, Spondylolysthesis, Surgical implant, Vertebral collapse, Other lesions. Any true spine radiographs with more than one of the types of above were not included in GAN training; there were 494 of these multiple abnormality type radiographs. Data augmentation for all GAN models included adaptive discriminator augmentation (parameter 0.6) (blit, geom, color, filter, noise, cutout) enabled. Both StyleGAN2–ADA and SpineGAN were trained for 4,200,000 images shown to the discriminator. StyleGAN2 is known to converge after 5,000,000 images are shown to the discriminator, and from qualitative observation of generated outputs, both StyleGAN and SpineGAN were trained until 4,200,000 images shown to the discriminator to limit the expensive computational cost. However, due to compute costs StyleGAN–ADA–MultiConditional was trained with only 1,200,000 images shown to the discriminator. StyleGAN2–ADA is known to take longer than 3 days to converge; however, as proof of concept, this paper did not train until convergence. All GAN training used batch size of 4, consistent with standard StyleGAN hyperparameters.

SpineGAN differs from StyleGAN2–ADA in its loss function. SpineGAN loss function uses the negative log sigmoid of the generated logits for the main generator loss function as is used in StyleGAN2–ADA; however, it also adds a term to this loss function. First, SpineClassifier was trained for 19 epochs with the above standard augmentation. After every generation in the SpineGAN main loop, SpineClassifier is applied to the generated images and Binary Cross Entropy loss term using the true labels as the conditional inputs is added to the main generator loss term for SpineGAN. This additional BCE term was weighted with an empirically determined 0.01 factor. This additional loss term was developed in order to provide knowledge of abnormality during GAN training. While in theory, a conditional GAN should learn both how to generate radiographs as well as condition them on abnormality, because spine radiographs differ in such few features between these conditions, it was hypothesized that including this extra loss term could accelerate the GAN’s training.

*Classifier Models*

For binary abnormality classification, the DenseNet121 model architecture was used with a linear layer, dropout (0.1), and sigmoid activation added to the end of the model. Weights were pretrained on Imagenet. Binary cross entropy loss with class weights was used as the loss function. Training continued for all classifiers for 30 epochs. This epoch number was arbitrarily selected without a validation dataset as it resulted in near-state-of-the-art performance and thus it was not necessary to computationally search to determine this hyperparameter.
