# Supplementary material for: Demonstrating the successful application of synthetic learning in spine surgery for training multi–center models with increased patient privacy
Source: Sci Rep. 2023 Aug 1;13:12481. doi: 10.1038/s41598-023-39458-y (PMC10393976; doi:10.1038/s41598-023-39458-y)
Supplement: Supplementary file 2 — Supplementary Information 2. [file 41598_2023_39458_MOESM2_ESM.docx]

**
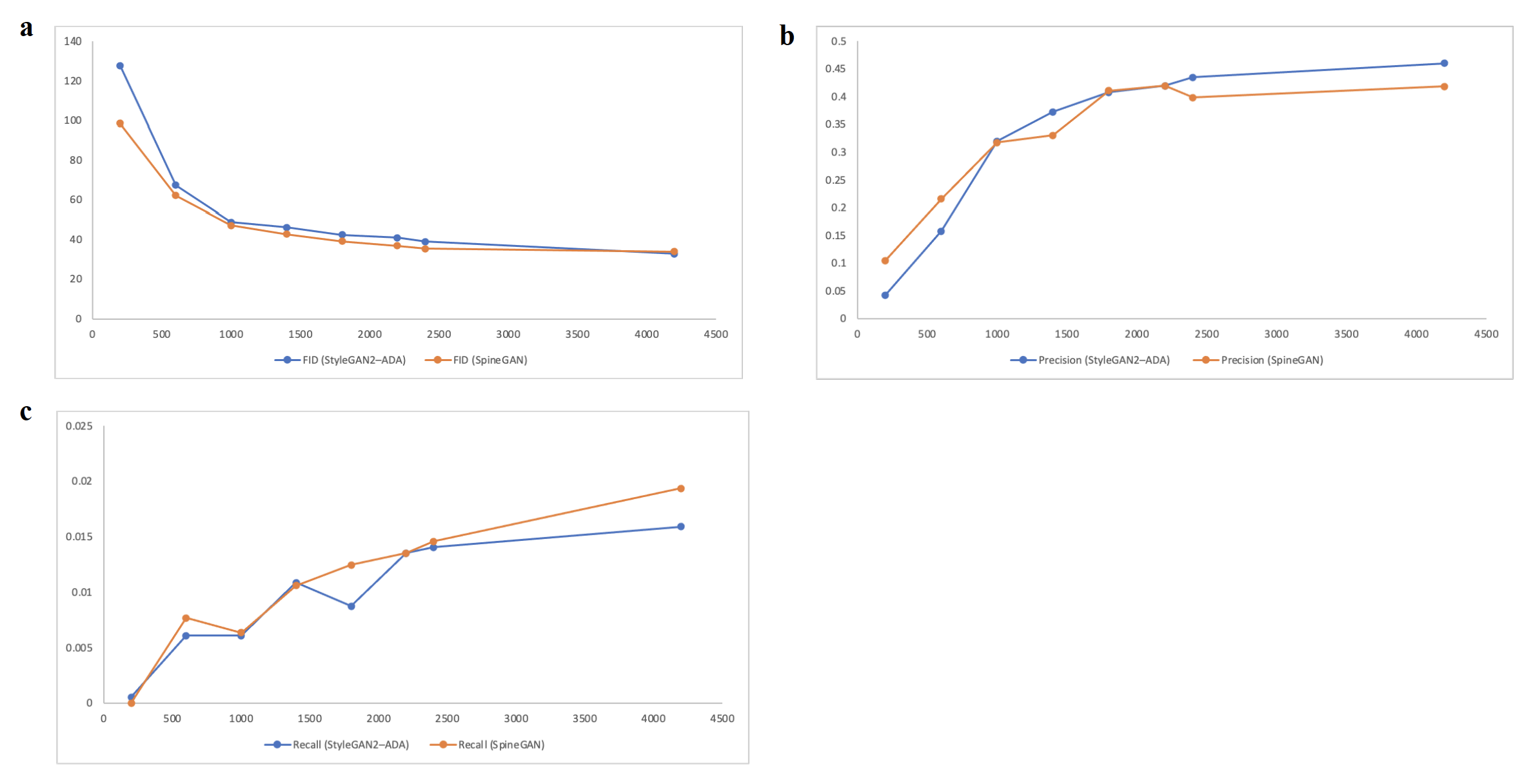
**

**Supplementary Figure 1**, GAN Performance by increasing training checkpoints (in thousands of images shown to the discriminator). SpineGAN differs from the traditional GAN by an additional domain loss term using a trained abnormality classifier. This domain loss term appears to accelerate training while resulting in similar (a. Frechet´ Inception Distance (FID), b. Precision) or greater (c. Recall) outcomes. Blue is SpineGAN and Orange is the traditional GAN.
